# Genetic characterization of *Toxoplasma gondii* in meat-producing animals in Iran

**DOI:** 10.1186/s13071-022-05360-1

**Published:** 2022-07-11

**Authors:** Afsaneh Amouei, Shahabeddin Sarvi, Azadeh Mizani, Mohammad Bagher Hashemi-Soteh, Saeid Salehi, Javad Javidnia, Seyed Abdollah Hosseini, Fateme Amuei, Ahad Alizadeh, Shafigheh Shabanzade, Sara Gholami, Ahmad Daryani

**Affiliations:** 1 Toxoplasmosis Research Center, Communicable Diseases Institute, Mazandaran University of Medical Sciences, Sari, Iran; 2 Department of Parasitology, School of Medicine, Mazandaran University of Medical Science, Sari, 4847191971 Mazandaran Iran; 3 Department of Parasitology, Pasteur Institute of Iran, Tehran, Iran; 4 Department of Clinical Biochemistry and Genetics, Faculty of Medicine, Mazandaran University of Medical Sciences, Sari, Iran; 5 Mazandaran Provincial Veterinary Department of Medical Sciences, Sari, Iran; 6 Department of Mycology, School of Medicine, Mazandaran University of Medical Science, Sari, Iran; 7 Department of Organic Chemistry, University of Mazandaran, Babolsar, Iran; 8 Metabolic Diseases Research Center, Research Institute for Prevention of Non-Communicable Diseases, Qazvin University of Medical Sciences, Qazvin, Iran; 9 Department of Parasitology and Mycology, School of Medicine, Shiraz University of Medical Science, Shiraz, Iran

**Keywords:** *Toxoplasma gondii*, Genotype, Diversity, Meat-producing animals, Mazandaran, Iran

## Abstract

**Background:**

The consumption of uncooked or undercooked food from infected intermediate hosts can result in *Toxoplasma gondii* infection in humans. However, few studies have investigated the genetic diversity of this protozoan parasite in Iran. The aim of the present study was to genetically characterize isolates of *T. gondii* from intermediate host animals in Mazandaran Province, Iran.

**Methods:**

Blood and heart tissue samples were collected from 204 ruminants, and brain tissue was collected from 335 birds. The prevalence of *T. gondii* infection in these samples was determined serologically using the modified agglutination test and by conventional PCR assays. Those PCR samples positive for *T. gondii* DNA and 13 DNA samples extracted from aborted fetuses in a previous study were genotyped with 12 genetic markers using the multilocus-nested PCR-restriction fragment length polymorphism (Mn-PCR–RFLP) technique.

**Results:**

Antibodies for parasites were found in 35.7% of the ruminant (39.1% of sheep and 26.4% of goats) samples and in 51.3% of the bird (100% of geese, 52.3% of free-range chickens and 46% of ducks) samples. Molecular detection by PCR of the repetitive 529-bp DNA fragment revealed contamination of 13.2% of ruminant (14.6% of sheep and 9.4% of goats) samples and of 9.6% of bird (11.1% of chickens, 5.7% of ducks and 0% of geese samples). The results from 30 DNA samples revealed five ToxoDB (genome database for the genus* Toxoplasma*) PCR–RFLP genotypes, including #1 (Type II), #2 (Type III), #10 (Type I), #27 and #48, with genotype #1 the most predominant.

**Conclusions:**

As evidenced by the results of this study, ruminants and birds are infected with *T. gondii* in the region, suggesting that they are likely to be involved in the transmission of *T. gondii* to humans through meat consumption. The identification of different genotypes may suggest a higher genetic diversity of this parasite in Mazandaran, reflecting local environmental contamination. These results have important public health implications for the prevention and control strategies of infection.

**Graphical Abstract:**

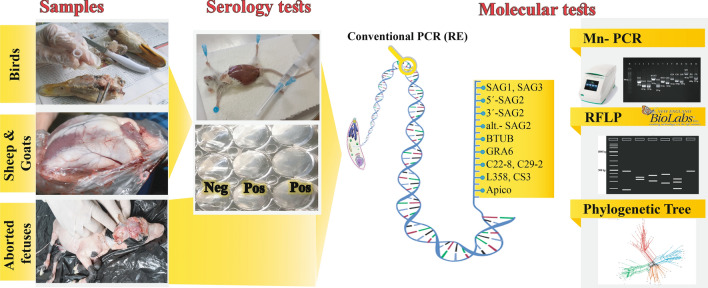

**Supplementary Information:**

The online version contains supplementary material available at 10.1186/s13071-022-05360-1.

## Background

*Toxoplasma gondii* is a zoonotic parasite with a worldwide distribution that infects warm-blooded vertebrate animals, including ruminants and birds [1]. The majority of human *T. gondii* infections occur via accidental ingestion of food and water contaminated with sporulated oocysts of *T. gondii* and the ingestion of raw or undercooked meat from *T. gondii*-infected intermediate hosts. Transplacental transmission of tachyzoites occurs from non-immune mothers during primary maternal infection [2]. Infection with this parasite leads not only to clinical signs in healthy animals, but can also result in abortion and neonatal mortality in several farm animals and even death in adult animals [3]. Since *Toxoplasma* is considered to be one of the most important food-borne pathogens, meat-producing animals serve as one of the main sources of human infections [4]. Therefore, it has widespread public health significance since it causes opportunistic zoonosis infections in people with compromised immune systems, such as those with acquired immunodeficiency syndrome (AIDS) [5].

The unique population structure of this parasite is unpredictably oligoclonal, and was initially grouped into three clonal lineage types (I, II, and III) with limited genetic diversity. Among these three main designations, Type II is predominant in North America and Europe [6, 7]. On the contrary, it has been reported that parasite isolates from some African and Central and South American countries have a high genetic diversity with no dominance of lineages [8–10].

Information on parasite population structure is important from a public health perspective; nonetheless, such data on Asian parasite populations is currently insufficient. Recent efforts have focused on genetically characterizing parasite isolates from different regions and hosts, providing new information on the genetic diversity and structure of *T. gondii* [11–13]. Nonetheless, little research has been conducted on the distribution of parasite genotypes in Iran and even fewer studies have focused on the genotypes circulating in animals. Food-producing animals are a major source of infection since they reflect specific geographical areas of parasitic zoonosis. In this study, the multilocus-nested PCR-restriction fragment length polymorphism (Mn-PCR–RFLP) technique was used to genotype isolates in intermediate hosts (i.e. ruminants and birds) collected from different areas in Mazandaran, Iran. To the best of our knowledge, we report here the first research to investigate the genotypes of *T. gondii* in meat-producing animals. Therefore, the present study aimed to examine this domain, elucidating possible sources and routes of infection in northern Iran.

## Methods

### Study area

All of the tissue samples used in the study were collected from Mazandaran districts since this region is one of the most important foci of the farming and poultry industry of Iran. The geographical locations and natural climatic conditions of the areas the samples were collected have been described in previous studies [14, 15]. Inhabitants in this area have a custom of consuming undercooked meat as a meal called ‘Iranian kebab’.

### Sampling protocol

This retrospective study was conducted on experimental samples (*n* = 204) obtained from the blood and hearts of ruminants (151 sheep and 53 goats) between October 2017 and April 2019. The investigations were conducted with the approval of the Animal Ethics Committee of Mazandaran University of Medical Sciences, Mazandaran, Iran (IR.MAZUMS.REC.94.1714). Permission to collect the biological samples at abattoirs was granted by the Chief Veterinary Officer of Mazandaran. Data related to each animal were documented for four independent variables, namely age, gender, animal species and region.

Sheep and goats of the study regions were slaughtered for human consumption at one of three abattoirs located in the western (62 animals), central (135 animals) and eastern (7 animals) regions of the province, respectively. The sampling was conducted using the data supplied by the Veterinary Officer and the abattoir manager. All of the animals were brought to the slaughter house by traders, who in turn had obtained them from farmers in the same area. In our study, sheep and goats, aged > 6 months, were selected randomly from among the slaughtered animals. Almost all slaughtered animals were male since female animals are normally kept for breeding purposes. All animals had been maintained predominantly in a semi-extensive type of management system (i.e. they were fed by grazing in communal natural grasslands during the daytime and kept in sheds at night). Data on sampling methods, the results of serological surveys of birds, and conventional PCR assays on aborted fetuses have been published in previous reports [14, 15].

Approximately 3–5 ml of blood was drawn directly from the jugular vein just before slaughter, and the heart of each animal was removed immediately after slaughter, placed in an individual zip-lock bag on ice packs, labeled appropriately and transported to the parasitology laboratory of Mazandaran University of Medical Sciences. Once in the laboratory, the sera were extracted from the blood samples, transferred to Eppendorf tubes and kept in a freezer at − 20 °C, and the collected hearts were kept refrigerated at + 4 °C until used. Different parts of the brain tissue were collected from 335 free-range birds, including 243 chickens, 87 ducks and five geese. The samples were labeled and stored in 70% ethanol until used for the molecular investigation.

### Serological analysis

The in-house modified agglutination test (MAT) was performed to detect *T. gondii*-specific IgG antibodies using an antigen prepared from formalin-fixed whole RH strain tachyzoites as described previously [16]. The sheep and goat sera were diluted twofold, starting with 1:10 and ending with 1:640 or higher. The cut-off titer of 1:20 was considered to indicate seropositive cases [16, 17].

### DNA extraction and conventional PCR assay

For the PCR assay, 50 g of heart tissue from each sheep and goat (free of fat mass and connective tissue) was ground into 1- to 2-cm-sized pieces and homogenized via a hand-held blender. A 100-ml aliquot of 0.9% saline was then added to the homogenized tissue, the mixture was homogenized again for 30 s and then the homogenate mixed with 250 ml of acid pepsin solution. After incubation at 37 °C for 1 h, the product was filtered immediately through two layers of cotton gauze and then centrifuged at 1200 *g* for 10 min. The digest was neutralized with 15 ml of 1.2% sodium carbonate solution before being centrifuged at 2000 *g* for 10 min [18]. A 2-ml volume of this homogenate was used for DNA extraction. For the assay on brain tissue, first, the entire sample of brain tissue collected from the birds was mixed and homogenized for 5 min, following which 100-mg samples were macerated using a mortar and pestle chilled with liquid nitrogen. The DNA extraction was performed using the DynaBio Tissue Kit (Takapouzist Co., Tehran, Iran) in accordance with the volumes used in the manufacturer's protocol. Finally, the purified nucleic acid sample was easily dissolved in 50 μl of sterile TE buffer (10 mM Tris–HCl, 1 mM EDTA), its concentration assessed by ultraviolet (UV) spectrophotometric absorbance at 260/280 nm and then the sample was stored at − 20 °C before further investigation.

The quality of the extracted DNA was verified by PCR using the host gene [i.e. receptor tyrosine kinase 2 (erbB-2)] as previously described [15].

*Toxoplasma gondii* molecular detection was performed by analyzing the DNA isolated from each sample using the PCR method with the amplification of the RE gene (200- to 300-fold repetitive sequence). The PCR assay was run in a 25-μl reaction volume consisting of 12.5 μl 2× Master Mix (Ampliqon, Odense, Denmark), 1 μl of purified DNA template, 0.6 μl of each PCR primer (10 pmol/μl; Bioneer, Daejeon, South Korea) and 10.3 μl of double-deionized water; the two primers used were Tox_4_ (forward: 5′-CGCTGCAGGGAGGAAGACGAAAGTTG-3′) and Tox_5_ (reverse 5′-CGCTGCAGACACAGTGCATCTGGATT-3′); fragment size: 529 bp. PCR amplification cycling consisted of pre-denaturation at 93 °C for 5 min, followed by 30 cycles at 93 °C for 30 s (denaturation), 55 °C for 30 s (annealing) and 72 °C for 30 s, and an extension step for 5 min (BioRad Laboratories, Hercules, CA, USA) [19]. The PCR fragments were separated by 1.5% agarose gel electrophoresis, and the PCR products were resolved by staining with a safe stain (CinaGen Co., Tehran, Iran) and visualized under a UV lamp on a transilluminator. In this study, RH strain (HBRC for *Toxoplasma*, Limoges University, France) was included as the positive control, and sterile distilled water was used as the negative control in all experiments.

### Genotyping analysis

Genotyping was performed by the Mn-PCR–RFLP method using 12 genetic markers (SAG1, 5′-3′ SAG2, alt-SAG2, SAG3, GRA6, BTUB, c22-8, c29-2, PK1, L358, Apico and CS3) as previously described in detail [10, 20]. The process of genotyping was carried out on 59 DNA samples isolated from sheep, goats and birds in this study, as well as on 13 DNA samples previously collected from aborted fetuses [15]. Parasite reference strains were also used in the genotyping process, including Type I (RH), Type II (PRU) and Type III (VEG), which were kindly provided by Dr. Marie-Laure Dardé at the University of Limoges, France.

The multiplex PCR reaction was performed in a final reaction volume of 25 μl consisting of 12.5 μl 2× Master Mix, 1.5 μl genomic DNA, 0.3 μM MgCl_2_ and 0.3 μM external primers of each gene marker in a single reaction. The PCR cycling regimen for this analysis consisted of an initial hot start step for 4 min at 95 °C, followed by 30 cycles of 30 s at 94 °C, 1 min at 55 °C and 2 min at 72 °C, with a final extension step for 5 min at 72 °C. The products of the first round were used as templates for the second round of PCR amplification by adding 25 μl of nuclease-free water (1:1). The nested PCR reaction was performed separately for each molecular marker in 25 μl of PCR mixture containing 12.5 μl 2× Master Mix, 1 μl diluted multiplex PCR products, 0.3 μM MgCl_2_ and 0.75 μl (10 pmol/μl) of each of the internal primers. This PCR cycling regimen consisted of an initial denaturation at 95 °C for 4 min, followed by 35 cycles at 95 °C for 30 s, at 60 °C for 1 min (BTUB and Apico markers: 58 °C), at 72 °C for 90 s, with a final extension at 72 °C for 3 min.

For the RFLP typing procedure, 5 μl of PCR amplified products was digested with the appropriate restriction enzyme(s) in a total reaction volume of 20 μl (New England Biolabs, Ipswich, MA USA). The restriction fragments were electrophoresed in a 3% agarose gel in 1× TBE buffer at 100 V for 1 h and the products then photographed. The typing data were analyzed, compared, and categorized according to the reference strain profiles in ToxoDB, a genome database for the genus* Toxoplasma* (http://toxodb.org/toxo/) [20]. Moreover, 20-μl samples of the purified PCR products from 19 different samples were sequenced for 12 genetic markers in both directions (forward and reverse) by the Pishgam Company (Tehran, Iran). In this study, the sequence of the obtained samples was edited, justified and aligned using Sequencher Tmv.4.1.4 software.

### Multilocus analysis by neighbor-joining clustering

The phylogenetic network was inferred using SplitsTree software (version 4.4) using the neighbor-joining algorithm between the genotype obtained in the present study and others isolated in previous research [21]. Multilocus PCR–RFLP typing data (with or without DNA restriction fragments) were coded for an allele of the locus with a combination of 0 and 1; thereafter, the genetic distances were estimated using the Tajima-Nei method.

### Statistical analysis

Statistical analysis was carried out in StatsDirect software version 2.7.2 (StatsDirect Ltd., Altrincham, UK) using a standard Chi-squared method with 95% confidence intervals (95% CI). The degree of agreement between two tests (serological tests and PCR assays) was explored using Cohen’s kappa coefficient (*k*). A *P*-value of < 0.05 was considered statistically significant.

## Results

### Sampling and prevalence analysis

The statistical population of this research (*n* = 204) consisted of 151 (74%) and 53 (26%) sheep and goats, respectively. Overall, the antibodies against *T. gondii* were found in 73 (35.8%) cases by MAT. In addition, the seroprevalence of the parasite was 39.1% and 26.4% in sheep and goats, respectively. The titers of positive sera were: 1:20 in five sheep, 1:40 in 11 sheep, 1:80 in 21 sheep, 1:160 in nine sheep, 1:320 in six sheep and ≥ 640 in seven sheep, and 1:20 and 1:40 in 0 goats, 1:80 in two goats, 1:160 in three goats, 1:320 in eight goats and ≥ 640 in one goat. Among this population, 34 (77%) samples had MAT titers of 1:160 or higher. No significant differences were found among the different regions and species (Additional file 1: Table S1). Seroprevalence had previously been verified in 51.3% of 335 birds (MAT titer ≥ 1:20 was seen in 100%, 52.3% and 46% of geese, free-range chickens and ducks, respectively) [14]. Subsequently, the DNA samples that were PCR-positive and 13 DNA samples (aborted fetuses) from a previous study were genotyped with 12 genetic markers using the Mn-PCR-RFLP technique.

### Detection in tissues

Table S2 summarizes the findings of the present study. The required tissue samples were obtained from all 204 hearts and 335 brains of birds screened for parasite DNA by conventional PCR. The PCR assays detected parasite DNA in 22 (14.6%) sheep, five (9.4%) goats, 27 (11.1%) free-range chickens and 5 (5.7%) ducks (Additional file 1: Table S2).

PCR-positive animals were almost all seropositive in this study. Positive PCR and negative MAT results were found in 14% of sheep (3/22), 0% of goats (0/5), 15% of free-range chickens (4/27) and 20% of ducks (1/5). However, Cohen’s kappa coefficient confirmed a slight degree of concordance between positive serology and positive PCR results (*k* = 0.18; Table 1).

**Table 1:** Correlation between serum MAT results and tissue PCR results

Species	No. tested	MAT	PCR		Kappa (95% CI)	*P*-value
			Positive	Negative		
Sheep	151	Positive	19	40	0.33 (0.19, 0.46)	< 0.001
		Negative	3	89		
Goat	53	Positive	5	9	0.45 (0.18, 0.72)	0.001
		Negative	0	39		
Free-range chicken	243	Positive	23	104	0.14 (0.07, 0.22)	< 0.001
		Negative	4	112		
Duck	87	Positive	4	42	0.06 (− 0.03, 0.15)	0.197
		Negative	1	40		
Geese	5	Positive	0	5	–	–
		Negative	0	0		
Total	539	–	59	480	0.18 (0.13, 0.24)	< 0.001

*CI* Confidence interval,* MAT* modified agglutination test

### Genotyping

Of the 72 DNA samples that tested positive for *T. gondii* by PCR, 30 (41.7%) were successfully amplified based on Mn-PCR primers (12 gene markers); the remaining 42 samples tested PCR-negative, which could explain the observed failure in obtaining sufficient amounts of parasite DNA. The DNA of animals was designated as TgShIr (sheep), TgGoIr (goat), TgBiIr (bird) and TgAbIr (aborted fetus). The 30 amplified *T. gondii* genotypes originated from sheep (*n* = 9), goats (*n* = 1), free-range chickens (*n* = 9), ducks (*n* = 1) and aborted fetuses (*n* = 10). Moreover, five genotypes were detected in the 30 DNA samples successfully genotyped, including ToxoDB PCR–RFLP genotype #1 (also known as clonal Type II lineage), #2 (also known as clonal Type III lineage), #10 (also known as clonal Type I lineage), #27 (also known as Type I variant) and #48 (also known as Type III variant), emphasizing the genetic variability of *T. gondii* in northern Iran. Genotypes #1 (13 animals, 43.3%) and #2 (11 animals, 36.7%) of *T. gondii* were identified in all species. Parasites with clonal Type II and III alleles were also predominantly detected in sheep and birds, respectively. Genotypes #10 (TgAbIr16, 18 and 19) and #27 (TgAbIr14 and 22) were identified in aborted fetuses; nonetheless, the genotype isolated from ducks belonged to genotype #48 (TgbiIr7). The results of genotyping the 30 parasite DNA samples at all multi-molecular markers are shown in Table 2. Among the PCR products, 19 different samples were subjected to sequencing using the forward and reverse primers mentioned in the Methods section. The genomic DNA sequences reported in this study were verified by aligning them with the relevant sequences associated with the parasite and subsequently submitted to the GenBank [accessions no. MH687540.1 (Apico), MH687541.1 (BTUB), MH687542.1 (GRA6), MH687543.1 (PK1), MH687544.1 (SAG3), MH704624.1 (GRA6), MH704645.1 (alt-SAG2), MH704646.1 (BTUB), MH704647.1 (C29-2), MH704648.1 (GRA6), MH704649.1 (CS3), MH704650.1 (3-SAG2), MH704651.1 (5-SAG2), MH704652.1 (C22-8), MH704653.1 (L358), MH704654.1 (SAG1), MH704655.1 (alt-SAG2), MH704656.1 (SAG3), MH704657.1 (GRA6)].

**Table 2:** Multilocus genotyping of *Toxoplasma gondii* isolates in animal samples from northern Iran

Isolate ID	Host	Genetic markers^a^												Toxo-DB genotype^b^
		SAG1	5′–3′-SAG2	alt-SAG2	SAG3	BTUB	GRA6	C22-8	C29-2	L358	PK1	Apico	CS3	
RH^c^		I	I	I	I	I	I	I	I	I	I	I	I	Reference (Type I)
PRU^c^		II	II	II	II	II	II	II	II	II	II	II	II	Reference (Type II)
VEG^c^		III	III	III	III	III	III	III	III	III	III	III	III	Reference (Type III)
TgShIr20	Sheep	II or III	III	III	III	III	III	III	III	III	III	III	III	#2
TgShIr23	Sheep	II	II	II	II	II	II	II	II	II	II	II	II	#1
TgShIr35	Sheep	II	II	II	II	II	II	II	II	II	II	II	II	#1
TgShIr37	Sheep	II	II	II	II	II	II	II	II	II	II	II	II	#1
TgShIr38	Sheep	II	II	II	II	II	II	II	II	II	II	II	II	#1
TgShIr43	Sheep	II	II	II	II	II	II	II	II	II	II	II	II	#1
TgGoIr132	Goat	II or III	III	III	III	III	III	III	III	III	III	III	III	#2
TgShIr168	Sheep	II	II	II	II	II	II	II	NA	II	II	NA	II	#1
TgShIr169	Sheep	II	II	II	II	II	II	II	NA	II	II	NA	II	#1
TgShIr174	Sheep	II or III	III	III	III	III	III	NA	III	III	III	III	III	#2
TgDuIr7	Duck	I	III	III	III	III	III	III	III	III	III	III	III	#48
TgCkIr9	Chicken	II	II	II	II	II	II	II	II	II	II	II	II	#1
TgCkIr26	Chicken	II or III	III	III	III	III	III	III	III	III	III	NA	III	#2
TgCkIr27	Chicken	II or III	III	III	III	III	III	III	III	III	III	III	III	#2
TgCkIr28	Chicken	II or III	III	III	III	III	III	III	III	III	III	III	III	#2
TgCkIr29	Chicken	II	II	II	II	II	II	II	II	II	II	NA	II	#1
TgCkIr30	Chicken	II or III	III	III	III	III	III	III	III	III	III	NA	III	#2
TgCkIr31	Chicken	II or III	III	III	III	III	III	III	III	III	III	III	III	#2
TgCkIr35	Chicken	II or III	III	III	III	III	III	III	III	III	III	III	III	#2
TgCkIr37	Chicken	II or III	III	III	III	III	III	III	III	III	NA	III	III	#2
TgAbIr3	Aborted fetus	II or III	III	III	III	III	III	III	III	III	III	III	III	#2
TgAbIr12	Aborted fetus	II	II	II	II	II	II	NA	II	II	II	NA	II	#1
TgAbIr14	Aborted fetus	I	I	I	I	I	I	I	III	I	I	I	I	#27
TgAbIr16	Aborted fetus	I	I	I	I	I	I	I	I	I	I	I	I	#10
TgAbIr18	Aborted fetus	I	I	I	I	I	I	I	I	I	I	I	I	#10
TgAbIr19	Aborted fetus	I	I	I	I	I	I	I	I	I	NA	I	I	#10
TgAbIr20	Aborted fetus	II	II	II	II	II	II	NA	II	II	II	NA	II	#1
TgAbIr22	Aborted fetus	I	I	I	I	I	I	I	III	I	I	I	I	#27
TgAbIr28	Aborted fetus	II	II	II	II	II	II	II	II	II	II	II	II	#1
TgAbIr30	Aborted fetus	II	II	II	II	II	II	II	II	II	II	II	II	#1

*NA* No amplification,* Toxo-DB* genome database for the genus* Toxoplasma*

^a^For details of markers, see [10, 20]

^b^Toxo-DB reference strains: #1 (Type II), #2 (Type III), #10 (Type I), #27 and #48

^c^Parasite reference strains: Type I (RH), Type II (PRU), Type III (VEG)

### Multilocus PCR–RFLP analysis of parasite genotypes obtained by phylogeny network

**Fig. 1:**
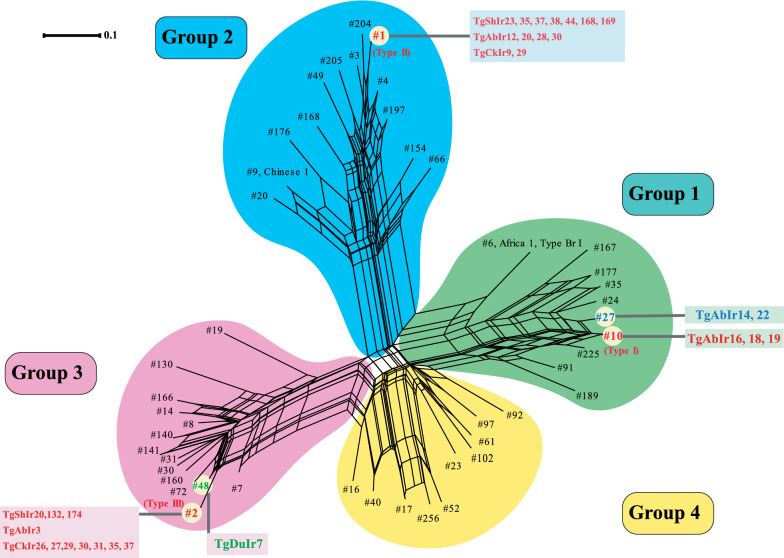
Phylogenetic network analysis of identified genotypes of viable *Toxoplasma gondii* isolates in animal samples from northern Iran (SplitsTree4 software). The genotypes closely related to Type I, II and III lineages are shown in green, blue, and purple circles, respectively. The genotypes of the fourth group are in yellow circles

A total of five RFLP genotypes were detected among the DNA extracted from each of the 30 samples and analyzed. The neighbor-net analysis was performed using the coded genotyping data from the 47 reference strains in the ToxoDB site. The results of the phylogenetic network, which was carried out using 11 multilocus RFLP markers plus one apicoplast genome, demonstrated that the majority of the representative strains were grouped in four genetic clusters (i.e. distinct clades and haplogroups). The neighbor-joining trees had three clonal types as references (I, II, and III), which were designated as groups 1, 2, and 3, respectively. The population structures of the fourth group explained the more complex Types I and III alleles. In the current study, phylogenetic analysis of animal isolates indicated that the samples could be classified into three phylogenies groups of 1, 2 and 3, with the majority of determined isolates in groups 2 (13/30) and 3 (12/30); the remaining five isolates were placed in group 1 (5/30), as presented in Fig. 1.

## Discussion

Farm animals are an economically significant commodity in many countries, making major contributions to milk, meat and dairy products and playing an important role in breeding [22–24]. In Iran, meat production from sheep, goats and birds has shown an increasing trend in recent years (See http://faostat.fao.org). Therefore, the presence of* T. gondii* in different tissues of livestock species highlights the potential importance of these animals as possible sources of *T. gondii* transmission to humans. Based on a systematic review and meta-analysis (1977–2012), the pooled seroprevalence of toxoplasmosis was reported to be 31% (range: 20–95%) and 27% (range 14.2–30%), respectively, among sheep and goats in Mazandaran Province [25]. In the current study, the results of the MAT on tissues from slaughtered sheep (39.1%) and goats (26.4%) were in close agreement with those of the earlier study (Additional file 1: Table S1). The worldwide prevalence of *T. gondii* among small ruminants varies widely across countries, in both sheep (minimum of 3% in China to a maximum of 94.8% in the USA) and goats (minimum of 7.8% in Spain to a maximum of 90% in Egypt) [26–29]. We hypothesized that many factors could be associated with this variation, such as geographical and climatic factors, sample size, age of animals, density of infected cats shedding oocysts, type of production or management system, access to contaminated feed and water, techniques used to diagnose infection, as well as the sensitivity–specificity of the testing kits used and their cut-off points. In the present study, semi-extensive rearing systems were predominant for herds, and seropositivity for toxoplasmosis antibodies was higher in sheep than in goats. Other related studies generally reported a higher prevalence in sheep, which may be explained by differences in feeding behavior, susceptibility to diseases and breeds. For example, sheep are mostly grazers and tend to consume the bottoms of grasses (greater exposure to sporulated oocysts); in comparison, goats are browsers and feed off the tops of plants and small trees [30–34].

The results of this study demonstrate that *Toxoplasma* DNA (Toxo-DNA) could be detected by PCR assay in the tissues of naturally infected animals since we detected Toxo-DNA in 27 heart samples from 204 small ruminants and in 32 brain samples from 335 birds. The prevalence rates of Toxo-DNA in the sheep, goats and birds analyzed in the present study were 14.6%, 9.4% and 9.6%, respectively (Additional file 2: Table S2), which are higher than those reported in studies performed in other countries, such as Poland (6.9%) [35], China (5.2%) [36] and India (2.3%) [37] in sheep, goats and birds, respectively, but lower than those reported previously in Iran [38] and in Tunisia (32.5%) [39] and Kenya (79%) [40] in sheep, goats and birds, respectively. Therefore, the findings of these studies illustrate the prevalence of viable parasites, whereas serological tests primarily detect chronic toxoplasmosis in animals [41]. It is worth noting that our positive PCR findings are not definitive in terms of estimating a true prevalence of infection since only one organ of each animal was selected for analysis. This study also found a slight correlation (*k* = 0.18) between serological and molecular approaches for the detection of infection; however, it used a larger size of the fragments (50 g) to extract and then evaluate DNA (Table 1). The primers TOX4 and TOX5 (a multi-copy repetitive 529-bp fragment) were used to detect the parasite due to their high sensitivity and specificity [19]. The repetitive 529-bp fragment is capable of detecting the limit of ≥ 1/50 of a parasite genome equivalent [42].

Since it is essential to understand the genotypes of parasites involved in infection for epidemiological investigations, and there was limited parasite DNA available in the biological samples, we used the Mn-PCR–RFLP assay with more genetic markers [43]. Nevertheless, two systematic review studies indicated that the genotypic diversity of animal isolates differ according to geographical and host origin [12, 13]. In the present study, DNA sequencing analysis of 19 isolates with 12 markers was completed successfully and although there were limitations, all of the sequencing data were in agreement with the RFLP results. The genotyping results in this study were based on Mn-PCR–RFLP (12 gene markers) and indicated that out of 30 parasite isolates from farm animals for human consumption and aborted fetuses in Mazandaran, 24 cases were PCR–RFLP genotypes #1 (known as Type II) and #2 (known as Type III), and the remaining six isolates were genotypes #10 (known as Type I), #27 and #48 (Table 2). These findings suggest that these genotypes may be common lineages circulating in this part of Iran. However, in earlier studies these genotypes were also detected in residents of northern Iran, indicating the genetic diversity and possible circulation of *T. gondii* genotypes in this area. The genotyping process in these studies identified four genotypes of parasite, including four genotypes (#1, #2, #10 and #27), in donors’ blood and six different genotypes, namely genotypes #1, #2, #10, #27, #35 and #48, in HIV-positive patients [44, 45]. In our study, the analysis of the isolates from sheep suggested that clonal Type II was overwhelmingly the predominant lineage in this region. The results of studies performed in Europe and the USA indicated that Type II strains are the most frequently identified genotype in humans and animals [12, 46, 47]. These findings are in line with those obtained in the present study. Nonetheless, the existence of these classic clonal lineages, especially Type II, is rare in South America [10]. Although a small number of isolates were studied in this research, *T. gondii* isolated from a goat presented genotype #2 and was clustered with Type III lineage (Table 2). The results of studies reported by Dubey et al. in the USA and by Mancianti et al. in Italy, with both groups investigating *T. gondii* among goats, found *T. gondii* of clonal Type III [48, 49].

The birds analyzed in this research were free-range animals that were also infected through ground feeding. Such free-range birds are considered one of the most important animal intermediate hosts in parasite epidemiology and they play a special role in *Toxoplasma* transmission to different species. The findings of isolates collected from these birds demonstrated that out of the 10 cases analyzed, all but three were genotype #2; the three exceptions were genotypes #1 (2 cases) and #48 (1 case) (Table 2). The predominance of lineage Type III over Type II isolates has been reported in studies conducted on diverse bird species in Egypt and Iran [50, 51]. Genotype #48 was identified and positioned close to clonal Type III by Bernstein et al. [52] in chickens, rabbit and rats in Argentina.

There is meager evidence on the genotypes of parasites circulating in aborted fetuses worldwide. The findings of the present study suggested that clonal Type II was the dominant lineage in aborted cases and was capable of causing spontaneous abortion [53]. The results of studies carried out from 1999 to 2002 demonstrated that all isolates were Type II based on one locus [54, 55]. This result was in accordance with the findings of a previous animal study conducted in Brazil [56]. In contrast to these results, there are reports of parasite strains named atypical genotypes from goat abortion cases in Argentina [57]. In the current research, one sample was infected with genotype #2, which was previously recognized from cases of ovine abortion in Ireland [53]. In the fetal analysis, isolated genotype #10 was a high pathogenicity strain, which has been mainly observed in Asia [20]. However, Type I was recognized in ovine aborted fetuses in Qazvin and Fars provinces, Iran [58, 59]. It is noteworthy that genotype #27, which is closely related to the clonal Type I of the TgAbIr14 and TgAbIr22, was recovered previously from a bird and cat in South America (Table 2) [10].

These data were supported by network analyses, which showed genetic diversity in several studied populations and identified three groups of ancestral types and related genotypes by SplitsTree analysis (Fig. 1). The comparison of identified genotypes among animal isolates revealed overlaps, except for genotypes #10, #27 and #48, the latter being identified in aborted cases and birds. The results of genotyping in this study suggested that Mazandaran province had an epidemic population structure of the parasite. Host-parasite interactions between resistant hosts and virulent *T. gondii* strains could likely render current population structures to the parasite. Regarding this, further research needs to be performed to gain more comprehensive knowledge in this domain.

## Conclusion

In general, the findings of this study indicate that the estimated prevalence of parasite infection among livestock is widespread in the study area. The DNA of *T. gondii* was detected in tissue samples from all tested animals (sheep, goats, birds and aborted fetuses), indicating that these animals might pose a risk to human health by transmitting human toxoplasmosis if their infected meat is eaten or raw meats are handled without proper hygienic procedures. To the best of our knowledge, this is the first report on the genotypes of *T. gondii* circulating in animals. The results of this study can be used for further epidemiological surveys since they reflect a specific geographical origin, elucidate possible sources and routes of parasite infection for humans and may have important implications for public health in Iran. Nevertheless, more research is needed to assess the pathological aspects of these genotypes in an animal model.

## Supplementary Information

**Additional file 1: Table S1.** Seroprevalence of *T. gondii* IgG antibodies by MAT in sheep and goats from northern Iran.

**Additional file 2: Table S2.** Detection of *T. gondii* DNA in livestock from northern Iran.

## Data Availability

The datasets used and/or analyzed during the current study are available from the corresponding author upon reasonable request.

## Abbreviations

DNA: Deoxyribonucleic acid
PCR: Polymerase chain reaction
MAT: Modified agglutination test
Mn-PCR–RFLP: Multilocus-nested PCR-restriction fragment length polymorphism
