# Supplementary material for: Genetic characterization of Toxoplasma gondii in meat-producing animals in Iran
Source: Parasit Vectors. 2022 Jul 11;15:255. doi: 10.1186/s13071-022-05360-1 (PMC9277799; doi:10.1186/s13071-022-05360-1)
Supplement: Supplementary file 1 — Additional file 1: Table S1. Seroprevalence of T. gondii IgG antibodies by MAT in sheep and goats from northern Iran. [file 13071_2022_5360_MOESM1_ESM.docx]

| Species | No. tested | No. positive (%) | N0. of positive sera with MAT titers of: | | | | | | Center region | | East region | | West region | | Statistics |
| --- | --- | --- | --- | --- | --- | --- | --- | --- | --- | --- | --- | --- | --- | --- | --- |
|  |  |  | 1:20 | 1:40 | 1:80 | 1:160 | 1:320 | ≥640 | No. | No. positive (%) | No. | No. positive (%) | No. | No. positive (%) |  |
| Sheep | 151 | 59 (39.1) | 5 | 11 | 21 | 9 | 6 | 7 | 97 | 44 (45.4) | 7 | 1 (14.3) | 47 | 14 (29.8) | P= 0.07  *X*^2^=5.11  df= 2 |
| Goats | 53 | 14 (26.4) | - | - | 2 | 3 | 8 | 1 | 38 | 11 (28.9) | - | - | 15 | 3 (20) | P= 0.51  *X*^2^=0.44  df= 1 |
| Total | 204 | 73 (35.8) | 5 | 11 | 23 | 12 | 14 | 8 | 135 | 55 (40.7) | 7 | 1 (14.3) | 62 | 17 (27.4) |  |

**Table S1** Seroprevalence of *T. gondii* IgG antibodies by MAT in sheep and goats from northern Iran
