# Supplementary material for: Genetic characterization of Toxoplasma gondii in meat-producing animals in Iran
Source: Parasit Vectors. 2022 Jul 11;15:255. doi: 10.1186/s13071-022-05360-1 (PMC9277799; doi:10.1186/s13071-022-05360-1)
Supplement: Supplementary file 2 — Additional file 2: Table S2. Detection of T. gondii DNA in livestock from northern Iran. [file 13071_2022_5360_MOESM2_ESM.docx]

**Table S2** Detection of *T. gondii* DNA in livestock from northern Iran

| Species | No. samples | No. (%) of identified infections |
| --- | --- | --- |
| Sheep | 151 | 22 (14.6) |
| Goat | 53 | 5 (9.4) |
| Free-range chicken | 243 | 27 (11.1) |
| Duck | 87 | 5 (5.7) |
| Geese | 5 | - |
| Aborted fetus [15] | 70 | 13 (18.6) |
